# Maternal Knowledge, Attitudes, and Practices Toward Complementary Feeding in Rural Bangladesh: A Cross‐Sectional Study

**DOI:** 10.1002/hsr2.72318

**Published:** 2026-04-08

**Authors:** Rajib Ul Islam, Morsheda Alam Mili, Sabkat Kamal, Labina Taher, Fariha Noshin, Mst Sabrina Moonajilin

**Affiliations:** ^1^ Department of Government and Politics Jahangirnagar University Savar Dhaka Bangladesh; ^2^ Department of Public Health and Informatics Jahangirnagar University Savar Dhaka Bangladesh; ^3^ Department of Urban and Regional Planning Jahangirnagar University Savar Dhaka Bangladesh; ^4^ Kelvin Grove State College Brisbane Queensland Australia; ^5^ Government College of Applied Human Science Dhaka Bangladesh; ^6^ Queensland University of Technology, Psychology and counselling Brisbane Australia

**Keywords:** child nutrition, complementary feeding, maternal knowledge

## Abstract

**Background:**

Complementary Feeding is essential for child growth and development, particularly between 6 and 24 months of age, when nutritional demands exceed what breast milk alone can provide. However, persistent gaps remain between maternal knowledge and actual feeding practices in many low‐ and middle‐income countries (LMICs). Inappropriate complementary feeding contributes significantly to child malnutrition in Bangladesh. This study examined the knowledge, attitudes, and practices (KAP) of mothers regarding complementary feeding in a rural area of Tangail, Bangladesh.

**Methods:**

A cross‐sectional survey was conducted among 160 mothers of infants aged 6–24 months. Data were collected using a structured questionnaire, guided by World Health Organisation (WHO) recommendations and previously validated KAP instruments.

**Results:**

Most mothers (93.1%) had introduced complementary feeding to their children, and 82.5% correctly identified the appropriate age for initiating. Only 66.9% practised adequate hygiene in food preparation, and 93.1% affirmed the importance of nutritious complementary food for proper growth and development. Homemade foods were preferred by 89.4% of mothers, though 16.3% introduced formula milk early. Attitudes toward feeding during illness were mixed. However, 60% of mothers reported offering extra food during illness, which aligns with WHO recommendations. Mothers with higher education and income demonstrated significantly more positive attitudes, and employed mothers and those in nuclear families showed significantly better complementary feeding practices.

**Conclusion:**

This study found while most mothers in rural Bangladesh know when to initiate complementary feeding, substantial gaps persist in hygiene and feeding practices. Increased community‐based and family‐centered nutrition education, as well as the integration of growth monitoring into maternal and child health services, are recommended to address observed practice gaps. Further research should explore barriers to applying knowledge in practice.

## 1 Introduction

Complementary feeding is defined as the systematic introduction of appropriate, safe, and nutritionally adequate foods alongside continued breastfeeding to meet the evolving nutritional needs of infants [1]. According to the World Health Organisation (WHO), exclusive breastfeeding is recommended for the first 6 months of life, after which breast milk alone becomes insufficient to support the infant's physiological growth and cognitive development. Infants require additional sources of energy and micronutrients to prevent nutritional deficits and growth faltering [2, 3]. The period between 6 and 24 months of age is widely recognized as a critical window of opportunity for promoting optimal growth, neurodevelopment, and long‐term health [4]. Failure to introduce complementary foods at the appropriate time, or the provision of foods that are nutritionally inadequate or hygienically unsafe, can result in a cascade of adverse outcomes, including stunting, micronutrient deficiencies, impaired cognitive function, increased risk of infection, and even mortality [5, 6]. It is well documented that the adverse effects of early nutritional deficiencies, particularly stunting, are largely irreversible [1, 7].

WHO has developed evidence‐based guideli principles for complementary feeding practices, which emphasize not only the nutritional content and consistency of food but also feeding behavior and hygiene. These include continued, on‐demand breastfeeding until 2 years of age or beyond; responsive feeding practices that respect the child's hunger and satiety cues; gradual increases in food quantity and diversity; appropriate meal frequency based on age; and maintaining safe food preparation and handling practices. WHO recommends 2–3 meals per day for infants aged 6–8 months, increasing to 3–4 meals with 1–2 snacks for children aged 9–23 months [3, 8].

Improper complementary feeding is associated with multiple health risks, including diarrhea, choking, food allergies, protein‐energy malnutrition (PEM), and long‐term developmental delays [9]. Timely and appropriate complementary feeding practices have been shown to reduce infant mortality, enhance immune function, and support age‐appropriate physical and mental development [3, 10]. Despite global emphasis on optimal complementary feeding, cultural beliefs, maternal knowledge, socioeconomic status, and caregiving practices remain significant barriers to implementation [11, 12, 13]. In many LMICs, including Bangladesh, maternal understanding of complementary feeding is shaped by intergenerational practices and local beliefs, which may not align with current nutritional recommendations [14, 15]. The responsibility of complementary feeding generally rests with mothers, whose capacity to adhere to best practices is often constrained by lack of information, economic limitations, or sociocultural norms [16, 17, 18].

Malnutrition, particularly undernutrition, remains one of the leading contributors to child mortality and morbidity worldwide [19, 20]. Globally, more than 2 billion people suffer from micronutrient deficiencies, with undernutrition contributing to approximately 45% of deaths among children under 5 years old. An estimated 2.6 million children die annually due to malnutrition, with stunting affecting around 160 million children globally. Vitamin A and zinc deficiencies alone are responsible for 157,000 and 116,000 child deaths per year, respectively [21, 22]. The increasing burden of non‐communicable diseases (NCDs) in Bangladesh places a significant strain on the healthcare system, both in terms of direct medical costs and indirect costs such as loss of productivity [23, 24, 25]. In Bangladesh, the burden of malnutrition is particularly severe. Over 450,000 children suffer from severe acute malnutrition, while more than 2 million experience moderate acute malnutrition [26, 27]. Anemia affects 52% of children under five, and 41% of children are stunted, one of the highest rates in South Asia. Moreover, 36% of children under five are underweight, while 16% are already overweight, indicating a troubling double burden of malnutrition [28, 29]. Among women of reproductive age, a quarter are underweight, and 15% are of short stature, increasing the risk of complications during childbirth and the likelihood of low‐birth‐weight infants [30, 31]. Anemia affects nearly half of all Bangladeshi women, largely due to inadequate dietary intake [32, 33]. Socio‐cultural norms, environmental conditions, and limited access to healthcare complicate motherhood in Bangladesh [34, 35].

The economic consequences of malnutrition are substantial, with estimated productivity losses exceeding US$1 billion annually in Bangladesh alone [36]. Although recent health sector efforts have contributed to modest improvements in child survival indicators, the overall burden remains high. The infant mortality rate declined from 28 per 1000 live births in 2016 to 21 in 2020, while under‐five mortality fell from 35 to 28 per 1000 live births during the same period [37]. Nevertheless, gender disparities persist; male infants continue to experience slightly higher mortality rates than females, and rural infants are more at risk than their urban counterparts [38, 39].

The study showed wide variation in KAP regarding complementary feeding. Inadequate practices regarding timing, dietary diversity, meal frequency, and hygiene continue to drive undernutrition [2, 40]. In Bangladesh, national and subnational analyses reveal mixed progress: persistent delays in the appropriate introduction of complementary feeding and limited dietary diversity, despite the recommended messages being widely known [41, 42]. This study aimed to assess the KAP regarding complementary feeding among mothers of young children in Tangail, Bangladesh, and to explore how socio‐demographic factors influence these components. The importance of promoting evidence‐based complementary feeding practices is both a public health imperative and a strategic investment in the country's human capital. Interventions aimed at improving maternal knowledge and community‐level feeding practices, particularly among socioeconomically disadvantaged groups, are critical to reversing child malnutrition and ensuring a healthier future for the next generation.

## 2 Methods

### 2.1 Study Design

This study employed a descriptive cross‐sectional survey design to assess the KAP of a specific population at a single point in time. Data were collected in 2022 from mothers of infants aged 6–24 months residing in Tangail district, Bangladesh. Cross‐sectional studies are particularly appropriate in public health research for generating baseline data on community practices and informing future interventions.

### 2.2 Study Area and Population

The study was conducted in Pakutia Union, located in Nagarpur Upazila of Tangail district, Bangladesh. This area was selected due to its mixed rural setting, accessibility, and relevance to the study population. The target population consisted of mothers with children aged 6–24 months, a critical period for complementary feeding and complementary feeding practices.

### 2.3 Sample Size and Sampling Procedure

A total of 160 mothers participated in the study. In practice, the accessible population for this specific group of mothers actively complementary feeding children in the defined age range within a rural community was relatively limited. Participants were selected using a convenience sampling approach, given the practical constraints and absence of a formal household registry for the target population. While this limits generalizability, it allowed for focused data collection in a defined and relevant setting.

### 2.4 Data Collection Tool

The questionnaire used in this study was developed based on a review of existing literature and WHO guidelines on infant feeding practices, particularly complementary feeding [3, 27, 42]. The items were structured to assess key dimensions of maternal KAP related to weaning. To ensure content validity, the questionnaire was reviewed by local experts in maternal and child health and community nutrition. Their feedback was incorporated to refine the clarity, cultural appropriateness, and relevance of the items. The instrument was first developed in English and then translated into Bengali by a bilingual researcher. A back‐translation was subsequently carried out by an independent translator to ensure linguistic equivalence and semantic consistency between the original and translated versions [43]. Before full data collection, a pilot test was conducted with five mothers who met the study's inclusion criteria but were not part of the final sample. This pretest helped to assess item clarity, comprehension, and completion time. Minor modifications were made based on participant feedback. Knowledge had 11 items scored 1 for correct and 0 for incorrect or “don't know” responses (range 0–13). Attitude had 6 items on a 3‐point Likert scale (0 for disagree, 1 for neutral, 2 for agree), summed to a score of 0–12 and converted to a percentage of the maximum. Practice was measured by 17 behavior items, each scored 0 or 1, with a total score range of 0–17. To assess internal consistency reliability, Cronbach's alpha coefficients were calculated for each of the KAP sections: knowledge section (13 items): *α* = 0.82, attitude section (6 items): *α* = 0.78, and practice section (17 items): *α* = 0.84. These values indicate acceptable to high internal consistency, suggesting that the questionnaire reliably captured the intended constructs (Supporting Information S1).

### 2.5 Data Collection

Data were collected via face‐to‐face interviews using a Bengali questionnaire. The lead author administered the survey to ensure clarity and consistency. During data collection, COVID‐19 precautions were strictly followed, including the use of masks and hand sanitizers by both interviewers and participants. Face‐to‐face interviews were chosen to maximize response rates and improve data quality, especially given the low literacy levels among some participants. This approach also helped minimize item non‐response and allowed for clarification of questions when needed.

### 2.6 Data Analysis

All data were coded and analyzed using IBM SPSS Statistics Version 25. Descriptive statistics, including frequencies, percentages, means, and standard deviations, were used to summarize participants' demographic characteristics and KAP responses. We assessed normality using the Shapiro–Wilk test. Several KAP scores were not normally distributed, so we used nonparametric tests: the Mann–Whitney U test for two‐group comparisons and the Kruskal–Wallis H test for more than two groups. For categorical variables, we used the *χ* ^2^ or Fisher's exact test, as appropriate. Spearman's rank correlation assessed relationships between continuous variables. All tests were two‐sided, with *α* = 0.05. All statistical results were considered statistically significant at a *p* value of < 0.05 [44].

## 3 Results

### 3.1 Socio‐Demographic Characteristics

Most mothers (37.3%) were aged 26–30 years, and the majority (85%) were housewives. Regarding educational status, over half of the mothers (52.5%) had completed Secondary School Certificate (SSC) level education, while only 1.9% had no formal education. Fathers' educational attainment was also highest at the SSC level (40%), and nearly half (49.4%) were engaged in business. The largest proportion of children was aged 12–18 months (39.3%), and the sample was evenly split by gender (51.9% female, 48.1% male). Most families (65.6%) had a total monthly income below BDT 20,000. A majority (55.6%) lived in joint families, and 66.9% had 1–5 family members (Table 1).

**Table 1:** Socio‐demographic characteristics of respondents (*N* = 160).

Characteristic	Frequency (*n*)	Percentage (%)
Age (years)		
18–25	52	32.5
26–30	60	37.5
31–35	30	18.8
≥ 36	18	11.2
Occupation		
Housewife	136	85.0
Service	8	5.0
Business	6	3.8
Others	10	6.2
Mother's education		
No formal education	3	1.9
Primary	26	16.2
SSC	84	52.5
HSC	25	15.6
Graduate or higher	22	13.8
Total monthly income		
< 20,000 BDT	105	65.63
21,000–35,000 BDT	35	21.88
36,000–50,000 BDT	14	8.75
> 50,000 BDT	06	3.80
Religion		
Islam	154	96.25
Hindu	06	3.75
Type of family		
Single	71	44.4
Joint	89	55.6

Abbreviations: HSC = higher secondary certificate, SSC = secondary school certificate.

### 3.2 Mothers' Knowledge Regarding Complementary Feeding

Most mothers (93.1%) had introduced complementary feeding to their children, and 82.5% correctly identified the appropriate age (6–8 months) for initiating complementary feeding. However, 16.9% believed that complementary feeding should begin earlier (4–5 months), indicating some knowledge gaps. Regarding the type of complementary feeding, 79.4% of mothers identified liquid foods as suitable for initiation, and 70.6% introduced cow's milk as the first liquid. In terms of semi‐solid foods, 63.75% reported feeding khichuri, followed by Cerelac (43.75%). Rice (66.88%) was the most introduced solid food. Family members (58.75%) were the most frequently cited sources of information about complementary feeding, followed by doctors (38.75%). About 38.1% of mothers fed their children three meals daily, and 29.4% fed four or more meals.

### 3.3 Mothers' Attitudes Toward Complementary Feeding

Most mothers (84.4%) agreed that breastfeeding alone is insufficient to meet an infant's nutritional needs after 6 months, and 93.1% affirmed the importance of nutritious complementary feeding for proper growth and development. However, 50% disagreed that commercial food is more nutritious than homemade food. Attitudes toward feeding during illness were mixed: 51.9% believed less food should be given during illness, while 50.6% believed the same amount should be maintained. Encouragingly, 60% of mothers reported offering extra food during illness, which aligns with WHO recommendations.

### 3.4 Complementary Feeding Practices Among Mothers

Most mothers (89.4%) preferred homemade food for complementary feeding. The most common reason for initiating weaning was the child reaching an appropriate age (57.5%). In 95% of cases, the mother was the primary person responsible for food preparation. About 91.3% of mothers prepared new food daily, and 94.4% prepared separate food for the child. Safe feeding practices were mixed. Although 99.4% of mothers reported washing their hands before feeding, only 67.5% boiled drinking water. Plastic utensils or feeding bottles were used by 85% of mothers. Only 21.9% reported using a growth monitoring chart. Table 2 illustrates descriptive statistics for knowledge, attitude, and practice scores. Although knowledge about the timing of complementary feeding initiation was high (82.5% correctly identified 6 months), specific hygiene behaviors (such as boiling drinking water, 67.5%) and use of growth monitoring (21.9%) were much less common. These findings suggest the presence of both structural and behavioral barriers to following recommended practices.

**Table 2:** Descriptive statistics for knowledge, attitude, and practice scores.

Variable	Mean (*M*)	SD	Minimum	Maximum
Knowledge score	7.08	1.58	3	10
Attitude score	3.75	1.08	1	5
Practice score	9.98	1.83	5	13

### 3.5 Association Between Socio‐Demographic Variables and KAP Scores

Table 3 illustrates the relationships between various socio‐demographic characteristics and mothers' KAP regarding complementary feeding. The analysis revealed several significant associations, underscoring how specific background factors influence maternal attitudes and behaviors in this critical area of child nutrition. Mothers possessing a graduate‐level education demonstrated significantly higher attitude scores toward complementary feeding (*H* = 13.89, *p* = 0.01), suggesting that higher educational attainment positively shapes maternal perceptions and readiness to adopt recommended complementary feeding practices. Similarly, families with a monthly income ranging above BDT 50,000 exhibited elevated attitude scores (*H* = 11.44, *p* = 0.01), indicating that economic stability may contribute to more favorable attitudes towards appropriate infant feeding. Furthermore, the sex of the child appeared to influence maternal attitudes, with mothers of girl children recording significantly higher attitude scores compared to those with boys (*U* = 2600.5, *p* = 0.03). This finding may reflect culturally nuanced perspectives on gender and caregiving practices. In terms of practice scores, mothers residing in nuclear family settings scored significantly higher (U = 2495.0, *p* = 0.02) than those from extended families, implying that family structure may affect the implementation of complementary feeding practices. Additionally, mothers engaged in business activities exhibited notably higher practice scores (*H* = 11.32, *p* = 0.01), suggesting that maternal employment, particularly in business, may empower mothers with greater autonomy or access to resources that facilitate better complementary feeding practices.

**Table 3:** Statistically significant associations between socio‐demographic variables and KAP scores.

Variable	KAP domain	Test used	Test statistic	*p* value
Mother's education	Attitude	Kruskal–Wallis H	13.89	0.01
Occupation	Practice	Kruskal–Wallis H	11.32	0.01
Family income	Attitude	Kruskal–Wallis H	11.44	0.01
Family type	Practice	Mann–Whitney U	2495.00	0.02
Child's gender	Attitude	Mann–Whitney U	2600.50	0.03

## 4 Discussion

This study investigated maternal KAP related to complementary feeding among mothers of infants. The findings provide important insights into the adequacy of complementary feeding behaviors in a rural Bangladeshi context and align with broader global and regional health promotion literature on child nutrition and maternal health. Most of the mothers in this study demonstrated basic knowledge about complementary feeding, including the recommended initiation age (6–8 months) and the types of appropriate complementary foods. Eighty‐two point five percent correctly identified the recommended age for introducing solid and semi‐solid foods, aligning with WHO guidelines [3]. This rate is comparable to studies conducted in urban Dhaka [26] and Pakistan [9], but higher than rural populations in Uganda, where knowledge levels remain below 60% [45]. However, the persistence of outdated practices, such as 16.9% of mothers initiating weaning before 6 months and a strong preference for cow's milk, raises concern. Early introduction of complementary feeding can expose infants to infections, allergies, and micronutrient deficiencies [1]. This suggests that while awareness may be improving, misconceptions about optimal feeding persist, possibly due to intergenerational influence or misinformation from informal sources such as family members.

Overall, maternal attitudes toward complementary feeding were moderately positive, particularly in acknowledging the nutritional importance of complementary feeding after 6 months. However, conflicting beliefs emerged regarding feeding during illness, an area consistently identified as a barrier to adequate child nutrition in South Asia [9, 31, 32]. While 60% of mothers reported increasing food intake during illness, nearly half believed that food should be restricted, contradicting WHO recommendations to increase nutrient‐dense fluid and food intake during illness to prevent malnutrition and dehydration. Education and income were significantly associated with more favorable attitudes, a finding consistent with studies that highlight maternal education as a key determinant of nutritional behavior [15, 17, 30]. Furthermore, the association between attitude scores and the child's gender (with higher scores among mothers of daughters) may reflect differing caregiving expectations or heightened concern for female children's well‐being in specific cultural contexts, though this requires further exploration.

Encouragingly, most mothers preferred homemade food and reported good hygiene practices, such as handwashing before feeding and preparing fresh food daily. These practices reflect positive behavior change and may be linked to recent public health campaigns around maternal and child nutrition in Bangladesh [15]. However, the widespread use of plastic bottles and utensils raises concerns about exposure to microplastics and improper sterilization, both of which are associated with gastrointestinal infections in infants [46]. The practice score was significantly higher among mothers engaged in business and those from nuclear families. This may suggest that mothers with more autonomy or fewer domestic constraints are better positioned to apply health knowledge in daily routines. Similar patterns have been noted in research from Saudi Arabia and Ethiopia, where family structure influences maternal agency in feeding decisions [47, 48]. Only 21.9% of mothers used growth monitoring charts, a critical tool for tracking developmental milestones and identifying malnutrition risk. This highlights a missed opportunity for integrating education about growth surveillance into community health services.

Knowledge does not automatically translate into practice. Common barriers include limited household resources, caregiving workload, strong family influence (particularly from elders), and conflicting advice from informal networks. In this sample, family members were the most frequently cited sources of information, which likely moderates maternal decision‐making and may explain why some evidence‐based behaviors, such as the use of growth monitoring charts, remain uncommon despite general awareness. The mixed attitudes toward feeding during illness reflect persistent misconceptions: nearly half believed that less food should be given during illness, whereas the WHO recommends increased intake of nutrient‐dense fluids and foods to prevent weight loss and dehydration. Programmatic responses should combine behavior change communication targeting mothers with community‐level engagement of elders and fathers, as well as improved access to routine growth monitoring through community health workers.

The findings of this study must be interpreted considering Bangladesh's broader nutritional challenges. The country continues to experience a double burden of malnutrition, with both undernutrition and emerging trends in childhood obesity [30]. While infant mortality and under‐five mortality rates have declined, the persistent rates of stunting (41%) and underweight (36%) signal ongoing inadequacies in early childhood nutrition. These findings are consistent with Bangladesh's national nutrition strategies and its ambitions under SDG‐2. Scaling up community nutrition education and strengthening growth monitoring at the primary care level can help achieve child nutrition targets. Policies should emphasize family‐centered counseling and low‐cost dietary diversification strategies for resource‐constrained households.

This study contributes to the growing evidence base showing that improving maternal complementary feeding knowledge and practices can directly influence these indicators. Individual‐level interventions (e.g., behavior change communication) must be reinforced by supportive environments, such as community‐based nutrition education, peer support groups, and access to low‐cost, fortified complementary feeding. Incorporating culturally sensitive education into antenatal and postnatal care can further strengthen complementary feeding practices among mothers with lower education levels.

## 5 Conclusion

Although general awareness of complementary feeding is high among mothers, persistent gaps in practice and hygiene indicate the need for targeted interventions. Community‐based health promotion, particularly through frontline health workers, and the inclusion of fathers and elders in nutrition education, may bridge these gaps. Additional research should investigate the barriers that prevent knowledge from being translated into practice.

## 6 Limitations

There are some limitations. First, the non‐probability sampling technique and relatively small sample size may limit generalizability. Second, responses may be subject to social desirability bias, particularly in hygiene‐related questions. Third, self‐reporting may introduce bias. Fourth, the cross‐sectional design does not capture changes over time.

## 7 Recommendations for Practice and Research

To improve complementary feeding practices and reduce childhood malnutrition:

- Targeted maternal education should be prioritized during immunization and maternal health visits.
- Community health workers should be trained to promote growth monitoring and dispel complementary feeding myths.
- Mass media and digital health platforms could support the dissemination of evidence‐based feeding messages in local languages.
- Future studies should employ longitudinal and qualitative designs to assess the long‐term impacts of maternal weaning practices on child growth and development.
- Increase access to structured training programs on infant nutrition for caregivers

## Author Contributions

**Rajib Ul Islam:** conceptualization, data curation, writing – review and editing. **Morsheda Alam Mili:** conceptualization, methodology, data collection, writing – review and editing. **Sabkat Kamal:** data curation, writing – review and editing. **Labina Taher:** data curation, writing – review and editing. **Fariha Noshin:** data curation, writing – review and editing. **Mst Sabrina Moonajilin:** conceptualization, methodology, and writing – original draft.

## Funding

The authors have nothing to report.

## Ethics Statement

The ethical aspects of this study were also reviewed and approved by the Biosafety, Biosecurity, and Ethical Review Board of Jahangirnagar University, Savar, Dhaka‐1342, Bangladesh [Ref No: BBEC, JU/M 2022/10]. All responses were anonymous, ensuring data confidentiality.

## Consent

All participants provided their informed consent to participate in the study after being informed about the purpose of the study.

## Conflicts of Interest

The authors declare no conflicts of interest.

## Transparency Statement

The corresponding author, Sabkat Kamal, affirms that this manuscript is an honest, accurate, and transparent account of the study being reported; that no important aspects of the study have been omitted; and that any discrepancies from the study as planned (and, if relevant, registered) have been explained.

## Supporting information


Supporting File

## Data Availability

The data can be obtained from the corresponding author upon request.
