# Supplementary material for: Maternal Knowledge, Attitudes, and Practices Toward Complementary Feeding in Rural Bangladesh: A Cross‐Sectional Study
Source: Health Sci Rep. 2026 Apr 8;9(4):e72318. doi: 10.1002/hsr2.72318 (PMC13062521; doi:10.1002/hsr2.72318)
Supplement: Supplementary file 1 — Supporting File [file HSR2-9-e72318-s001.docx]

**Item-Level Knowledge, Attitude, and Practice Responses (N = 160)**

**A. Knowledge Items**

| **Knowledge Item** | **Response** | **n** | **%** |
| --- | --- | --- | --- |
| Correct first feed after birth | Breastmilk | 94 | 58.8% |
| Mothers who breastfed the baby | Yes | 126 | 78.8% |
| Correct initiation age of complementary feeding | 6–8 months | 132 | 82.5% |
| Correct reason for starting complementary feeding | Appropriate age of child | 88 | 55.0% |
| Correct type of food to introduce first | Liquid food | 127 | 79.4% |
| Knowledge of safe first liquids | Cow’s milk / formula (non-harmful liquids) | 140 | 87.5% |
| Semi-solid food options known | Khichuri | 102 | 63.8% |
| Solid food options known | Rice | 107 | 66.9% |
| Identified appropriate information source | Doctor/health worker | 64 | 40.0% |
| Correct feeding frequency per day | 3 or more times | 108 | 67.5% |
| Correct fruits for complementary feeding | Pomegranate / Orange / Banana | 149 | 93.1% |

**B. Attitude Items**

| **Attitude Item** | **Response** | **n** | **%** |
| --- | --- | --- | --- |
| Breastmilk alone is not enough after 6 months | Agree | 135 | 84.4% |
| Nutritious complementary food is essential | Agree | 149 | 93.1% |
| Family foods are healthier than commercial foods | Disagree with “commercial is more nutritious” | 80 | 50.0% |
| Feeding during illness – avoid reducing food | “No” to reducing food during illness | 77 | 48.1% |
| Feeding during illness – same amount acceptable | “Yes” | 81 | 50.6% |
| Feeding during illness – extra food recommended | “Yes” | 96 | 60.0% |

**C. Practice Items**

| **Practice Item** | **Practice** | **n** | **%** |
| --- | --- | --- | --- |
| Preferred complementary food | Homemade | 143 | 89.4% |
| Started complementary feeding at correct time | Appropriate age | 92 | 57.5% |
| Mother prepares child’s food | Yes | 152 | 95.0% |
| Prepares fresh food daily | Yes | 146 | 91.3% |
| Prepares separate food for child | Yes | 151 | 94.4% |
| Did not face difficulty starting CF | Yes | 132 | 82.5% |
| Child did not fall sick after starting CF | Yes | 134 | 83.8% |
| Started family foods at 6–8 months (not correct, but reported) | Yes | 146 | 91.3% |
| Safe water source used | Tube well / filtered / boiled | 154 | 96.3% |
| Boils water for drinking (safe practice) | Yes | 52 | 32.5% |
| Uses hygienic utensils (not plastic feeder) | “No” to plastic feeder | 24 | 15.0% |
| Handwashing before preparing/feeding | Yes | 159 | 99.4% |
| Uses soap for handwashing | Yes | 133 | 83.1% |
| Measures child's height/weight | Yes | 120 | 75.0% |
| Uses growth chart | Yes | 35 | 21.9% |
| Completed EPI vaccination | Yes | 66 | 41.3% |
| Continuing EPI vaccination | Yes | 88 | 55.0% |
